# Application of Multi-Omics Techniques in Aquatic Ecotoxicology: A Review

**DOI:** 10.3390/toxics13080653

**Published:** 2025-07-31

**Authors:** Boyang Li, Yizhang Zhang, Jinzhe Du, Chen Liu, Guorui Zhou, Mingrui Li, Zhenguang Yan

**Affiliations:** 1 State Key Laboratory of Environmental Criteria and Risk Assessment, Chinese Research Academy of Environmental Sciences, Beijing 100012, China; lby1969648319@163.com (B.L.); liuchen--@outlook.com (C.L.); zhougr2024@163.com (G.Z.); 202431470033@mail.bnu.edu.cn (M.L.); 2 Research Institute for Environmental Innovation (Tianjin Binhai), Tianjin 300450, China; zhangyz_hky@163.com; 3 School of Marine Science and Engineering, Qingdao Agricultural University, Qingdao 266109, China; djz@qau.edu.cn

**Keywords:** multi-omics, aquatic organisms, toxic effects, molecular mechanism

## Abstract

Traditional ecotoxicology primarily investigates pollutant toxicity through physiological, biochemical, and single-molecular indicators. However, the limited data obtained through this approach constrain its application in the mechanistic analysis of pollutant toxicity. Omics technologies have emerged as a major research focus in recent years, enabling the comprehensive and accurate analysis of biomolecular-level changes. The integration of multi-omics technologies can holistically reveal the molecular toxicity mechanisms of pollutants, representing a primary emphasis in current toxicological research. This paper introduces the fundamental concepts of genomics, transcriptomics, proteomics, and metabolomics, while reviewing recent advancements in integrated omics approaches within aquatic toxicology. Furthermore, it provides a reference framework for the implementation of multi-omics strategies in ecotoxicological investigations. While multi-omics integration enables the unprecedented reconstruction of pollutant-induced molecular cascades and earlier biomarker discovery (notably through microbiome–metabolome linkages), its full potential requires experimental designs, machine learning-enhanced data integration, and validation against traditional toxicological endpoints within environmentally relevant models.

## 1. Introduction

**Figure 1:**
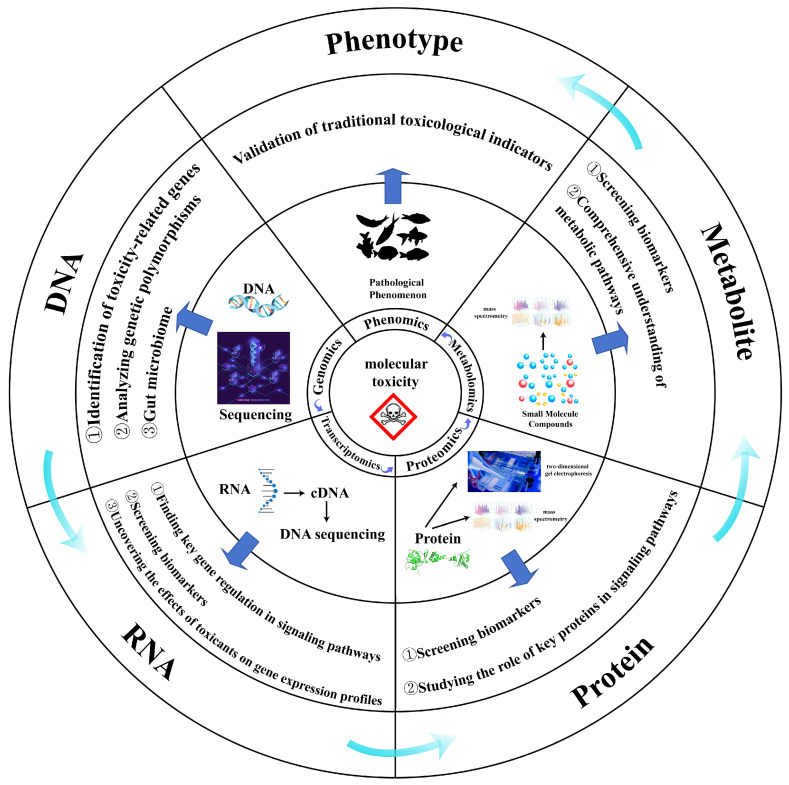
Integrated multi-omics framework in aquatic toxicology: pathway from molecular perturbations to phenotypic responses. Pollutants elicit toxicity through sequential molecular perturbations along the central dogma axis—from genomic alterations to metabolic dysregulation—culminating in adverse phenotypic outcomes. This cascade is interrogated by layer-specific omics technologies: genomics identifies toxicity-associated genetic variants and host–microbiome dynamics; transcriptomics pinpoints differentially expressed genes responsive to contaminant exposure; proteomics screens mechanistic biomarkers and characterizes functional protein networks; and metabolomics ultimately delineates biochemical pathway disruptions underlying toxic effects.

The accelerating pace of global industrialization has led to the unprecedented release of anthropogenic pollutants into aquatic ecosystems, posing significant threats to biodiversity and human health through bioaccumulation and trophic transfer [1]. Ecotoxicology research aims to decipher the mechanistic actions of environmental contaminants—including parent compounds and transformation byproducts—on biological systems [2]. This entails establishing dose–response relationships, identifying toxicity biomarkers, and providing scientific foundations for evidence-based environmental governance. Aquatic ecosystems sustain critical biogeochemical cycles while providing essential services including water purification, nutrient cycling, and habitat provision [3]. Their biotic components span functional groups from vertebrates to planktonic communities, each exhibiting differential vulnerability to pollutants [4]. Traditional ecotoxicological assessments relying on isolated physiological or biochemical endpoints face limitations in elucidating system-level toxicity mechanisms. Computational advances now enable transformative applications of omics technologies (genomics, transcriptomics, proteomics, metabolomics; Figure 1) and their integration [5,6,7]. These high-throughput approaches systematically characterize molecular responses across biological hierarchies, revealing functional perturbations in exposed organisms [6]. The current research landscape of multi-omics applications in environmental toxicology remains predominantly confined to single- or dual-omics approaches. Existing review articles in this field have primarily focused on omics investigations of specific pollutant categories, while conspicuously lacking systematic analyses at either the omics modality or organismal taxonomic level—such as comparative evaluations of the prevalence of particular omics techniques or cross-species differences in omics application patterns. This review systematically evaluates conventional multi-omics integrations applied to common aquatic organisms over the past five years (2019–2024). Through comprehensive literature interrogation, we aim to provide an understanding of the application and problems of multi-omics in different species and explore the current issues that need to be addressed for the development of multi-omics in the field of environmental toxicology.

## 2. Common Single-Omics Method

### 2.1. Genomics

Genomics is a technology that involves collectively characterizing all genes in an organism, conducting quantitative research, and comparing different genomes, mainly focusing on the structure and function of the genome. As the “source” of the “central law”, DNA plays a very important role as the cornerstone of the organism, and genomic analysis enables the acquisition of DNA sequence data via sequencing technologies [8]. The application process of genomics involves firstly extracting DNA from appropriate tissues and purifying it, performing high-throughput sequencing after preprocessing steps (e.g., fragmentation, amplification, etc.), and subsequently cleaning, integrating, and analyzing the downstream data, stitching together the genome sequences of the species, and extracting information (functional annotations) from these massive datasets through data mining. The most widely used technology on the market today is second-generation sequencing, and the innovativeness of this technology stems from the use of sequencing-by-synthesis (SBS) instead of traditional Sanger sequencing [9]. Commercial applications of second-generation sequencing are dominated by Roche’s 454 GS FLX platform, Life Technologies’ SOLiD platform, and Illumina’s HiSeq and MiSeq platforms [10]. In contrast, third-generation sequencing (TGS) technologies, developed in recent years, eliminate the need for PCR amplification and effectively reduce false positives through iterative sequencing. Current TGS platforms enable the direct sequencing of DNA or RNA molecules without requiring reverse transcription or preamplification steps, thereby bypassing read length limitations and achieving full-length sequencing. Furthermore, these technologies concurrently allow for the detection of genomic modifications such as DNA methylation [11,12]. Among the four mainstream TGS platforms currently available, the most widely adopted are Pacific Biosciences’ (PacBio), (Menlo Park, CA, USA) Single-Molecule Real-Time (SMRT) sequencing and Oxford Nanopore Technologies’ (ONT), (Oxford, UK) single-molecule nanopore sequencing (Table A1).

### 2.2. Transcriptomics

Transcriptomics involves the systematic capture and analysis of the complete repertoire of RNA transcripts present within a cell or tissue at a defined temporal point, encompassing messenger RNAs (mRNAs), transfer RNAs (tRNAs), ribosomal RNAs (rRNAs), and non-coding RNAs (ncRNAs). This approach facilitates the investigation of differential gene expression profiles across distinct tissues or experimental conditions, thereby enabling the identification of pivotal genes implicated in pathological alterations. Current methodological paradigms in this field predominantly encompass two principal technologies: microarray hybridization and RNA sequencing (RNA-seq). While both techniques enable transcriptome characterization, they diverge significantly in their operational principles and prospective applications [13].

Microarray technology employs designed chips with fluorescently labeled cDNA probes of known sequences covalently immobilized on a solid substrate, enabling large-scale transcriptome analysis through hybridization with thousands of predefined probes. This methodology, however, presents inherent limitations: it requires prior knowledge of target sequences and demands substantial quantities of template RNA for reliable detection. In contrast, RNA sequencing (RNA-seq) is performed by sequencing cDNA libraries derived from reverse-transcribed RNA, thereby providing quantitative measurements of diverse RNA species, including mRNAs, small RNAs, and non-coding RNAs [14]. While microarray analysis permits the precise quantification of expression profiles for predetermined gene sets, RNA-seq leverages high-throughput sequencing platforms to achieve the comprehensive profiling of transcriptomes with base-level resolution, capturing both known and novel sequence information [15].

To determine the locations and functions of sequences obtained by sequencing in the genome, it is first necessary to compare them with the reference genome to look for differentially expressed genes (DEGs). GO functional analysis and KEGG pathway enrichment analysis are performed for such genes. GO (https://www.geneontology.org/ (accessed on 29 July 2025)) is an internationally standardized gene function classification system that can be divided into three ontologies, named molecular function (MF), cellular component (CC), and biological process (BP). The Kyoto Encyclopedia of Genes and Genomes (KEGG) (https://www.genome.jp/kegg/ (accessed on 29 July 2025)) is a public database for pathway deciphering, which can be used to label differentially expressed genes in signaling pathways, facilitating the search for upstream and downstream genes [16].

Recent advancements in transcriptomics, both in technological innovation and practical application, have demonstrated significant potential for molecular-level investigations in environmental toxicology (Table A2). For instance, single-cell transcriptomics (scRNA-seq) enables sequencing at the individual cell level, facilitating a deeper understanding of the differential impacts of pollutants across specific tissues [17]. This advancement has been exemplified in reproductive toxicology studies using zebrafish (*Danio rerio*), where scRNA-seq revealed pollutant-induced disruptions in germ cell development and sex hormone signaling pathways [18]. Spatial transcriptomics (ST), compared to conventional transcriptomic technologies, enables the acquisition of gene expression profiles with spatial resolution directly from intact tissue sections while preserving the original physiological context, achieving subcellular localization [19]. This capability facilitates the establishment of correlations between transcriptomic alterations and the intracellular microenvironment. Recent advancements in NanoString’s nCounter platform have enhanced its utility in molecular profiling, particularly through its amplification-free workflow and compatibility with low-quality or low-abundance samples, making it a robust tool for the differential expression analysis of both mRNAs and miRNAs [20].

### 2.3. Proteomics

Proteomics primarily focuses on investigating alterations in protein expression across diverse biological samples. In aquatic toxicology research, mass spectrometry (MS) is a core technique for protein identification (Table A3). Liquid chromatography–mass spectrometry (LC-MS) has become the mainstream method due to its high sensitivity and compatibility with complex biological samples. Among these, matrix-assisted laser desorption/ionization time-of-flight mass spectrometry (MALDI-TOF) is suitable for the rapid screening of large-molecular-weight proteins, while Orbitrap mass spectrometry, with its ultra-high resolution (>100,000 FWHM) and sub-femtomolar detection limits (e.g., 7 fmol for lysozyme in fish gill tissue), significantly enhances the identification of low-abundance proteins [21]. However, the high salt content in marine samples can reduce the ionization efficiency, necessitating desalination steps such as the use of peptide desalting columns to mitigate signal suppression.

Protein separation techniques are crucial for subsequent MS analysis. Gel-based methods, such as two-dimensional polyacrylamide gel electrophoresis (2D-PAGE), offer high-resolution separation based on the isoelectric point and molecular weight but suffer from low recovery rates for hydrophobic and membrane proteins [22]. In contrast, non-gel-based methods like multidimensional liquid chromatography (MudPIT) enhance the peak capacity and improve the detection of low-abundance proteins, especially in samples with salt concentrations exceeding 10 mM [23].

Quantitative proteomics methods include both labeled and label-free approaches. Labeled methods, such as Tandem Mass Tag (TMT) or iTRAQ, use isotopic labels to enable the simultaneous quantification of multiple samples, offering high accuracy but at a higher cost [24]. Label-free methods, such as label-free quantification (LFQ), allow for unlimited sample throughput and reduced costs but may suffer from poor reproducibility due to retention time drift and high missing value rates. In aquatic toxicology applications, labeled methods combined with data-independent acquisition (DIA) such as SWATH-MS are effective in analyzing protein pathway changes under pollutant exposure, while LFQ is more suitable for large-scale longitudinal studies [25].

### 2.4. Metabolomics

Metabolomics constitutes a systematic approach to the quantitative profiling of the complete repertoire of metabolites and their dynamic concentrations within a biological system. This methodology enables the precise delineation of the biochemical status in cells, tissues, or organs, while providing mechanistic insights to elucidate the functional roles of genes. Through deciphering interconnections among metabolic networks, metabolomics facilitates the integrative analysis of biological regulation, thereby advancing the systems-level comprehension of organismal physiology and pathophysiological states [26]. Distinct from other omics technologies, metabolomics specifically focuses on small-molecule compounds (<1500 Da) that serve as co-regulatory factors and signaling mediators for most proteins in biological systems. The inherent chemical stability of metabolites renders them optimal indicators for the monitoring of organismal responses to environmental perturbations. Contemporary metabolomics is broadly categorized into untargeted and targeted approaches: untargeted metabolomics aims for the comprehensive detection of metabolites within biological matrices, while targeted metabolomics focuses on predefined compounds with known biochemical relevance [27]. A standardized metabolomics workflow comprises five critical phases: (1) sample collection from biological specimens (e.g., urine, blood, saliva, tissue extracts, or biopsy specimens); (2) preprocessing, involving pH adjustment, concentration normalization, and matrix removal to minimize analytical interference; (3) metabolite analysis using separation–detection platforms (e.g., liquid chromatography–mass spectrometry) optimized for small-molecule resolution; (4) data processing, incorporating noise reduction, peak alignment, normalization, and multivariate statistical analyses; (5) biological interpretation through metabolite database matching (HMDB, KEGG, METLIN) to identify dysregulated signaling pathways and putative biomarkers, ultimately elucidating the toxicological mechanisms of environmental contaminants [28]. Current metabolomics approaches predominantly rely on mass spectrometry (MS) or nuclear magnetic resonance (NMR) spectroscopy. However, these methods are constrained by the requirement for diverse extraction protocols to achieve comprehensive metabolome coverage and remain susceptible to interference from isomeric and isotopic species. Ion mobility spectrometry–mass spectrometry (IMS-MS) offers a promising approach to optimizing metabolomic analyses. This technology enables expanded metabolome coverage, the improved resolution of isomeric compounds, enhanced signal-to-noise ratios, and the augmented detection of low-abundance ions [29]. IMS-MS acts like a molecular filter—it quickly sorts complex environmental mixtures, making pollutants easier to detect while reducing background noise from soil or water samples. The advent of hyperpolarized nuclear magnetic resonance (NMR) techniques offers a promising avenue to address the inherent sensitivity constraints of conventional NMR-based metabolomics approaches [30].

## 3. Multi-Omics Integrated Analysis Platform

**Figure 2:**
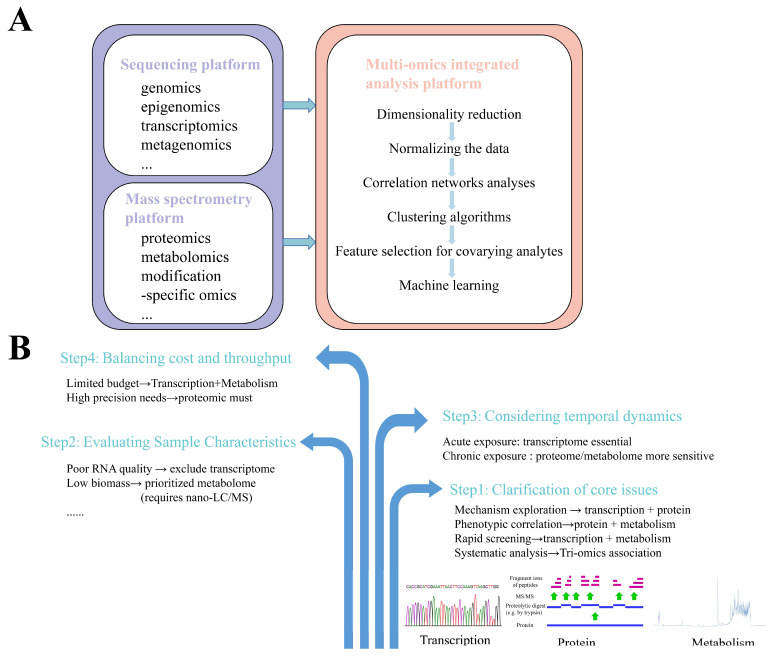
Multi-omics integrative analysis. (**A**) The general workflow for multi-omics integrative analysis. Sequencing platforms (genomics, epigenomics, transcriptomics, metagenomics) and mass spectrometry platforms (proteomics, metabolomics, modification-specific omics) feed into multi-omics integration featuring dimensionality reduction, data normalization, correlation networks, clustering algorithms, covariate-aware feature selection, and machine learning modeling. (**B**) Multi-omics selection strategies. Four-phase workflow: (1) core issue specification; (2) sample characteristic assessment; (3) temporal dynamics modeling (exposure duration/sampling intervals); (4) cost–throughput optimization (technology selection balancing resolution and resources).

Although the application of single-omics technology can enable the screening of biomarkers and provide biological explanations for corresponding toxicological mechanisms, it cannot fully capture comprehensive information about altered biological processes or systematically identify and interpret the specific toxic mechanisms of pollutants/compounds on organisms, including key events and molecules in adverse outcome pathways (AOPs). In contrast, multi-omics integration approaches combine data from different omics layers to enable the systematic analysis of organisms, thereby improving the understanding of signaling pathway dynamics and facilitating predictions about living systems. Multi-omics technologies also provide robust data support for systems biology research. By integrating data across multiple levels, they allow the joint analysis of hierarchical changes in organisms induced by chemical exposure, deepen insights into inter-level relationships, and offer rational foundations for mechanistic hypotheses [31]. The multi-omics technology platform primarily consists of three components: (1) a sequencing platform based on sequencing technologies, which supports omics such as genomics, epigenomics, transcriptomics, and metagenomics; (2) a mass spectrometry platform based on mass spectrometry technologies, supporting omics including proteomics, metabolomics, and modification-specific omics; (3) a multi-omics integrated analysis platform that combines sequencing and mass spectrometry data [32]. Among these, the multi-omics integrated analysis platform is the critical component determining the validity and rationality of joint multi-omics data analysis. The general workflow for multi-omics integrative analysis is illustrated in Figure 2A. Through appropriate analytical workflows and algorithms, multi-omics data can be clustered and functionally annotated. Bulk data from genomic, transcriptomic, proteomic, and metabolomic layers are processed within a unified integrative analysis software platform, including normalization, comparative analysis, and correlation analysis, to establish data correlations across molecular layers. Multi-omics integration tools can be categorized into data-driven analysis tools and knowledge-based analysis tools, with some online analysis platforms also available. Common integrative analysis tools are listed in Table 1. When conducting a multi-omics experimental design, the selection of an organizational technique should be made with reference to a number of factors (Figure 2B). For example, acute exposure experiments need to rely on the rapid response properties of the transcriptome to capture immediate changes in gene regulation, while chronic exposure experiments utilize proteomics and metabolomics to better resolve the cumulative effects.

Currently, challenges persist in the integration of multi-omics data. Heterogeneity frequently arises from multi-omics datasets acquired through distinct technologies. For example, transcriptomic and proteomic data exhibit divergent dynamic ranges and distribution patterns, attributable to variations in normalization protocols, analytical pipelines across omics platforms, and spatiotemporal discrepancies [33]. Furthermore, metabolomic datasets may contain missing values (zero-inflation) due to instrument detection limits, necessitating rigorous imputation strategies [34] and outlier detection during multi-omics data harmonization [35]. Notably, while RNA sequencing generates abundant transcript-level information, the technical complexity and temporal demands of proteomic and metabolomic profiling often result in annotation biases and elevated noise levels. Consequently, the overwhelming informational load from transcriptomics risks obscuring biologically meaningful signals in proteomic and metabolomic datasets. Optimal sample size requirements vary substantially across omics modalities, as statistical reliability is governed by the false discovery rate (FDR), which inversely correlates with the number of measured entities (transcripts/proteins/metabolites). Thus, multi-omics experimental designs must adhere to modality-specific sample size thresholds [36]. Tarazona developed a computational framework for the estimation of optimal sample sizes in multi-omics studies, addressing critical challenges in statistical power calculations [37]. Their methodology systematically evaluates critical parameters across sequencing- and non-sequencing-based omics platforms, yielding MultiPower—an open-source computational framework for the optimization of experimental designs.

Recent advances in artificial intelligence (AI) have profoundly transformed multi-omics data integration paradigms, addressing longstanding challenges in dimensionality reduction, heterogeneous data fusion, and predictive biomarker discovery. Modern AI frameworks—particularly deep learning (DL), graph neural networks (GNNs), and federated learning—now dominate cutting-edge platforms by enabling the end-to-end analysis of high-dimensional datasets while preserving the biological context. For instance, architectures such as ConcatAE (concatenated autoencoders) and MOFA+ (multi-omics factor analysis) leverage variational autoencoders to disentangle shared and dataset-specific latent factors, effectively resolving batch effects and missing data biases, which are common in aquatic toxicology studies [38]. Notably, GNNAI (GNN-derived representation alignment and integration), introduced in 2025, employs graph neural networks to align features across transcriptomic, proteomic, and metabolomic layers while identifying biomarker interactions under incomplete data scenarios—a critical capability for ecotoxicogenomic investigations, where full data coverage is rare [39].

**Table 1:** Common integrative analysis tools.

Tool	Core Function	Accessibility	Aquatic Model Suitability	Reference
Data-Driven Approaches				
mixOmics	Multi-omics integration and dimensionality reduction	R package	Supports transcriptome–metabolome integration	[40]
MOFA	Multi-omics factor analysis	Python/R	Supports time-series exposure experiments	[41]
OmicsNotebook	Cloud-based multi-omics integration	Web platform	Dedicated aquatic toxicology module	NA
SNF	Clustering and classification Similarity network fusion	R package	Supports transcriptome–metabolome integration	[42]
Knowledge-Based Approaches				
clusterProfiler	KEGG/GO enrichment analysis	R package	Supports model organism pathways	[43]
ReactomePA	Reactome pathway analysis	R package	30% improved coverage of fish signaling pathways	[44]
Cytoscape	Biological network visualization	Desktop	Supports microbe–host interaction networks	[45]
Pathview	Multi-omics pathway mapping	R/Bioconductor	Supports model fish and non-model species	[46]
Online Platforms				
Galaxy	Aquatic toxicology workflows	Cloud	Fish transcriptomics	[47]
OmicsNet	Multi-omics network analysis	Web platform	Supports aquatic toxicity biomarker networks	[48]
KNIME	Graphical workflow design	Desktop	Requires custom aquatic biology plugins	NA

## 4. Application of Multi-Omics in Ecotoxicological Studies of Aquatic Organisms

**Figure 3:**
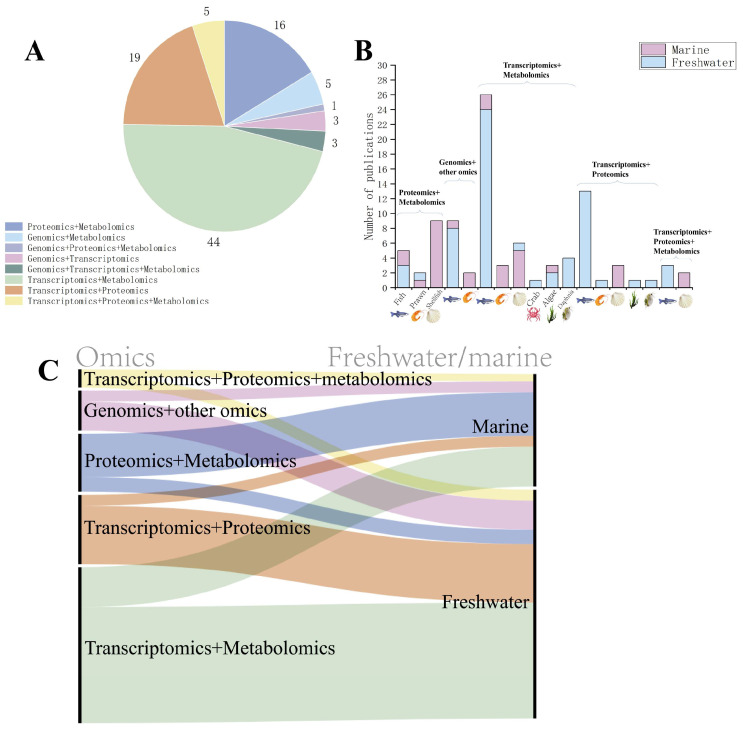
Literature related to multi-omics. (**A**) Current status of the application of multi-omics in the field of aquatic toxicology. In aquatic toxicology studies employing multi-omics approaches, transcriptome–metabolome integration represents the most prevalent combination. This is followed by tandem transcriptome–proteome and proteome–metabolome analyses. (**B**) Current status of multi-omics applied to different species. Current aquatic toxicology research employs five principal multi-omics integration schemes: ① proteomics + metabolomics, ② genomics + other omics, ③ transcriptomics + metabolomics, ④ transcriptomics + proteomics, and ⑤ transcriptomics + proteome + metabolomics (triple-omics). Implementation analysis across major taxonomic groups—fish, prawn, shellfish, crab, algae, and daphnids—reveals fish and shellfish as the predominantly adopted models, with crustaceans showing moderate application, while algal and daphnid studies represent emerging frontiers. (**C**) Linkages between research and applications of different multi-omics in freshwater or marine fields as shown by Sankey diagrams for literature analysis. In freshwater species, transcriptome–metabolome (green) integration represents the predominant multi-omics strategy in aquatic toxicology research. Conversely, proteome–metabolome (blue) integration emerges as the most frequently employed approach in marine organisms.

Thus far, biomarkers derived from omics approaches have been progressively integrated into environmental monitoring and risk assessment frameworks in aquatic biological research. For instance, the OECD Test Guidelines recommend incorporating transcriptomic biomarkers (e.g., HSP70 and CYP1A gene expression) as sublethal effect indicators to evaluate the early toxicity of pollutants such as pesticides and heavy metals. Additionally, ISO 23893-3 explicitly designates acetylcholinesterase (AChE) activity as a protein-level biomarker for organophosphate pesticide exposure [49]. Furthermore, ISO/TC 147/SC 5 is currently deliberating the inclusion of multi-omics data into standardized monitoring systems [50]. Much of the current research in the field of toxicology is moving towards multi-omics analyses to provide a comprehensive analysis of the mechanisms of toxicity at different molecular levels. Figure 3 shows the search results for “multi-omics”, “genomics”, “transcriptomics”, “proteomics”, “metabolomics”, “aquatic organism”, and “toxicology” in the Web of Science database to retrieve the literature data. Currently, transcriptome and metabolome or proteome analyses are more widely used, while microbiome technology applying genomic principles is also gradually being paid attention to in the field of toxicology and analyzed in conjunction with other omics. In the field of aquatic organisms, omics are gradually being emphasized and used in toxicological studies of a variety of organisms, including fish, shrimp, shellfish, crab, algae, trematodes, and many other marine or freshwater organisms. Most of the current research has focused on freshwater organisms. The most applied research programs are using transcriptomes and metabolomes.

The substantial disparity in the application frequency of different multi-omics approaches can be attributed to their respective strengths and limitations, as well as distinct problem-solving capabilities. The following sections will elaborate on the technical advantages and applicable scenarios of various omics integration methodologies. The combined application of proteomics and metabolomics focuses on elucidating direct correlations between functional phenotypes and metabolic regulation, particularly in investigating pollutant-induced alterations in protein activity and their associated fluctuations in metabolic products. This integrated approach demonstrates potential for revealing metabolic reprogramming driven by post-translational modifications (PTMs). For instance, Wang employed this “bottom-up” strategy to identify bioactive ligand-binding proteins, revealing SOD3 and GPX4 as key targets of benzo(a)pyrene (BaP)-induced hepatotoxicity through the mechanistic characterization of their regulatory networks [51]. Notably, proteins and metabolites in field-collected specimens may be better preserved over extended periods compared to RNAs. The integrated analysis of transcriptomics and metabolomics is frequently employed for the rapid screening of toxicological mechanisms and biomarker discovery, particularly in examining gene expression responses under acute exposure conditions, coupled with early-stage metabolic perturbations. This methodological synergy offers cost-effective advantages for mechanistic exploration, given that transcriptomic and metabolomic profiling incur substantially lower costs compared to proteomic analyses. Furthermore, such multi-omics integration proves particularly valuable for time-series dynamic analyses, enabling the systematic delineation of the temporal precedence in transcriptional regulation versus delayed metabolic adaptations. The integrated application of transcriptomic and proteomic profiling is frequently employed to investigate the decoupling phenomena between genetic expression and protein synthesis, with a particular focus on elucidating environmental contaminants’ disruptive effects on epigenetic regulation mechanisms [52]. The integrative application of transcriptomics, proteomics, and metabolomics constitutes a methodological cornerstone in systems toxicology, enabling the systematic construction of exposure–response continua from molecular initiating events (transcriptome-level perturbations) through key biological processes (proteome-level modifications) to adverse outcomes (metabolome-level dysregulation). This tri-omics paradigm facilitates the multidimensional assessment of emerging contaminants’ toxicodynamics, encompassing transcriptional interference networks, protein–ligand interaction landscapes, and systemic metabolic disruption profiles.

### 4.1. Combination of Proteomics and Metabolomics Analysis

Current research on proteomics–metabolomics integration remains limited, predominantly focusing on toxicological investigations in marine bivalves. In 2013, Wu’s team pioneered the combined application of proteomic and metabolomic approaches to integrate complementary omics datasets that directly characterized metabolic pathway perturbations, aiming to enhance the analytical precision [53]. Their multi-omics analysis revealed that arsenite exposure at environmentally relevant concentrations under hyposaline conditions induced energy metabolism dysregulation, evidenced by upregulated proteins including ATP synthase, succinyl-CoA synthetase, and nucleoside diphosphate kinase, with corresponding metabolic alterations in succinate and ATP levels. In the same year, using the same methodology, they found that BDE 47 (a polybrominated diphenyl ether) caused disturbances in energy metabolism in mussel Mytilus galloprovincialis and is sex-specific [54]. In 2016, the group investigated the developmental toxicity of cadmium and arsenic through exposure experiments and omics analyses of marine mussel larvae, and they found that As (III) had a significant effect on the cytoskeleton and cellular structure, that As (V) affected a number of key enzymes involved in energy metabolism and related to cellular development, and that cadmium induced oxidative stress, cellular damage, and disturbances in nucleic acid metabolism [55,56]. In 2018, Chen found that benzo(a)pyrene (BaP) caused teratogenicity, mutagenesis, and carcinogenicity in the pearl oyster Pinctada martensii by inducing signaling, transcriptional regulation, cell growth, stress responses, and energy metabolism [57]. The above studies provide a basis for the mechanisms behind the toxic effects of pollutants, but there are some problems with their multi-omics approach, and it is not reasonable to directly analyze data from different levels due to the heterogeneity and complexity between these omics data. Proper data analysis requires the application of different fusion strategies based on different data structures. In 2021, Dumas used multi-block modeling to objectively evaluate the most relevant data blocks by combining partial least squares regression-based Monte Carlo UVE (MCUVE-PLS) and consensus orthogonal partial least squares discriminant analysis (OPLS-DA) to more accurately find the most relevant signaling pathways [58]. In this way, they found that the induction of autophagy was closely related to carbamazepine’s mechanism of action, as well as the destabilization of lysosomal membranes and the enzymatic overactivity of peroxisomes. Moreover, in 2023, they found that multiple pharmacologically active compounds were able to produce a superimposed effect that led to disorders of energy metabolism, fatty acid degradation, protein synthesis, and degradation, as well as inducing endoplasmic reticulum stress and oxidative stress signaling pathways [59]. In addition to this, combined proteomic and metabolomic analyses exist in fish and shrimp [60,61,62].

In toxicological studies in freshwater, researchers have also gradually turned their attention to the proteome and metabolome, and, due to the early maturity and ease of use of the transcriptome, most of the early researchers conducted their studies through the transcriptome. However, now that proteomic and metabolomic technologies are maturing, more research will turn to proteomics and metabolomics because they are more closely aligned with the metabolic state in an organism. In 2017, Chen was the first to study changes in the expression of proteins and the profiles of metabolites in the liver of the small freshwater fish *Danio rerio* (zebrafish), which were investigated after long-term exposure to environmentally relevant concentrations of microcystin-LR (MC-LR) [63]. Moreover, in 2018, Le Manach used the medaka fish (*Oryzias latipes*) as a study species [64]. Together, these two studies reveal that microcystins induce dysbiosis in the fish liver through the production of bioactive peptides, including energy and steroid metabolism and protein synthesis, ultimately inducing toxicological effects in the fish liver. Procambarus clarkii, a globally invasive species, was used as a study species in freshwater shrimp research. Proteomics and metabolomics indicate that, even with high adaptability and tolerance, oxidative stress and mitochondrial dysfunction can be induced by the introduction of toxic metal elements into the water column, leading to alterations in glycolysis and lipid metabolism [65].

In environmental toxicology studies utilizing joint proteomic and metabolomic analyses, mussels emerge as the most frequently employed model species. This prevalence stems from the inherent synergy between the technical requirements of multi-omics approaches and the biological characteristics of mussels. Both proteomics and metabolomics demand substantial tissue quantities for reliable analysis. Mussels fulfill this requirement, as sufficient tissue (particularly from digestive glands and gills) can be readily obtained for the concurrent extraction and analysis of both proteomes and metabolomes. Furthermore, the relatively mature research foundation surrounding mussel biology facilitates the anchoring of observed protein/metabolite alterations to established biological processes, such as detoxification, immune responses, and stress responses. Finally, the low mobility of mussels contributes to a relatively stable metabolic baseline under specific environmental conditions. This stability enhances the detection sensitivity for pollutant-induced deviations from baseline in omics profiles.

### 4.2. Combination of Gut Microbiome and Other Omics Analysis

In environmental toxicology research, genomics provides a distinct perspective that differs from that of transcriptomics. Its primary applications include identifying susceptibility genes and genetic polymorphisms; elucidating the mechanisms and characteristics of environmental mutagenesis; conducting epigenomic studies; investigating host–microbiome interactions; and studying populations’ adaptive evolution. Currently, in aquatic toxicology, host–microbiome interaction research represents the most extensively applied genomic approach. Currently, the gut microbiome is emerging as an omics tool, being incorporated into aquatic ecotoxicology research and conceptually falling under the domain of metagenomics. The integration of gut microbiome analysis with multi-omics approaches represents an emerging paradigm, with methodological frameworks only coalescing within the past quinquennium. Current applications predominantly focus on freshwater fish models, although limited studies extend to marine teleosts and crustaceans. Notably, all existing gut microbiome–metabolome correlation studies remain confined to freshwater organisms. A seminal 2021 study pioneered the combined application of gut microbiome profiling and metabolomics in aquatic toxicology, examining dioxin-like pollutant effects [66]. Their work revealed that PCB126 exposure induces the dysbiosis-mediated disruption of bile acid and vitamin metabolism along the gut–liver axis, establishing a microbial–host metabolic crosstalk as a critical determinant in toxicological responses. Subsequent investigations have expanded this approach to zebrafish (*Danio rerio*), medaka (*Oryzias latipes*), and rare minnow (*Gobiocypris rarus*) models, elucidating the toxicity mechanisms of microcystins, microplastics, and cyclopropoxypyrrolizin (CPZ), with a particular emphasis on intestinal pathophysiology [67,68,69,70]. These studies consistently demonstrate that microbial community shifts precede metabolomic alterations, likely reflecting direct luminal interactions between xenobiotics and enteric microbiota. This temporal precedence positions the microbiome as a critical interface in pollutant toxicity cascades. In addition to this, since 2023, studies have been conducted to reveal the toxicogenic mechanisms of pollutants by linking the gut microbiome with the transcriptome and metabolome. Pei exposed adult zebrafish to local Sri Lankan drinking water and collected multiple omics data through the microbiome, transcriptome, and metabolome [71,72]. They found that metabolites involved in fatty acyl and glycerophospholipid classes were associated with zebrafish gut-dominant facultative bacteria and DEGs such as egln3, ca2, jun, slc2a1b, and gls2b. Comprehensive multi-omics analysis based on the context of host physiology in samples of renal injury of different pathological grades suggests significant changes in pathways such as calcium signaling and necrosis. Their research study has informed the subsequent evaluation of biomarkers needed for kidney disease. Yu also applied three omics to zebrafish exposure experiments and successfully revealed that low-dose and long-term sulfamethoxazole (SMZ) exposure leads to neurotoxicity and is strongly associated with alterations in the gut microbiome [73]. In summary, the advantage of applying multi-omics techniques containing gut microorganisms lies in the ability to take into account the interactions and effects between different organs of an organism, such as the mechanism of toxicant action in the gut–brain-axis, which provides a viable means of evaluating the toxic effects of pollutants as a whole, rather than in individual tissues or organs.

Currently, research on marine organisms is scarce and at an early stage. Most are only combined microbiome and transcriptome analyses, exploring alterations in biomarkers and the interaction networks of species after pollutant exposure. For example, Wang studied changes in the gut–hepatic axis in marine fish under zinc exposure [74]. Zhou found that changes in gene expression profiles associated with microplastic-induced neurological diseases and movement disorders in sea urchins were associated with changes in the intestinal microbiota [75]. Fu et al. found that transcriptome alterations induced by environmentally relevant concentrations of thiamethoxam, a nicotinic insecticide, correlated differently with different species of gut microorganisms, with a positive correlation with *Shimia* and a negative correlation with *Pseudoalteromonas* [76]. Research in this field still requires further studies to reveal the interactions between gut microbes and host metabolism and the specific mechanisms.

In multi-omics studies incorporating the microbiome, freshwater species are more frequently utilized. This likely stems from terrestrial-sourced pollutants—such as pesticides, antibiotics, and microplastics—being predominantly discharged into freshwater systems via agricultural runoff and wastewater effluents. Consequently, freshwater organisms serve as first-line sentinels for contaminant exposure. Positioned at mid-trophic levels in aquatic food webs, their contaminant bioaccumulation and microbiome dysbiosis pose direct human health implications through trophic transfer. Furthermore, integrating gut microbiome analysis with other omics technologies enables the establishment of a tripartite nexus linking host genetics, microbial community function, and metabolic phenotypes. This integrated approach elucidates mechanisms of interorgan toxicity transmission mediated by pollutants while leveraging the heightened sensitivity of microbiome profiling to identify early-warning biomarkers.

### 4.3. Combination of Transcriptomics and Metabolomics Analysis

Transcriptomics is often used in the study of molecular mechanisms of toxicology due to its mature development and low sequencing costs, while metabolomics is the fastest omics to reflect the environments of cells (including the nutritional states of cells, the effects of drugs and environmental pollutants, and other external factors) and can be used to analyze the effects of pollutants on organisms in the most intuitive manner. Thus, the combination of the two has naturally become the focus of research. Empirical analyses indicate that approximately 50% of multi-omics investigations in aquatic toxicology prioritize this dual-omics framework, reflecting its unique capacity to bridge molecular pathways with phenotypic manifestations.

Freshwater ecotoxicology research predominantly employs fish models, with zebrafish constituting nearly 50% of experimental organisms due to their evolutionary conservation and genomic homology with humans [77]. The reason is that the most perfect fish model organism is the zebrafish, whose genes are more homologous to humans, and the gene element profiles of zebrafish can be obtained by comparing its genome sequences with the clearer genome sequences of humans, mice, and so on [78]. In 2009, Williams’ team integrated transcriptomic and metabolomic data for the first time in the field of aquatic toxicology [79]. The effects of 1,2:5,6-dibenzanthracene (DbA) on the metabolic pathways of the stickleback (*Gasterosteus aculeatus*) were preliminary determined by changes in gene expression and metabolites, which provided ideas for the development of subsequent omics research. In 2017, Tauler’s team analyzed the transcriptomes and metabolomes of zebrafish embryos exposed to bisphenol A by using multivariate chemometric modeling and standard data analysis tools [80]. By integrating the results of the two omics, the reliability of the relevant molecular biomarkers searched for was improved, and the regulatory pathways responsible for the toxicity-induced changes in BPA in zebrafish embryos were further identified. Subsequently, the use of zebrafish for combined transcriptomic and metabolomic analyses has become increasingly common—for example, the use of zebrafish embryos to study the developmental toxicity of environmental pollutants such as plasticizers, endocrine drugs, biocides, microplastics, etc. [81,82,83] and the use of adult zebrafish to study tissue damage and metabolic disorders associated with pollutants such as pesticides, plastic additives, and other pollutants [84,85,86]. Notably, the study of joint transcriptome–metabolome analysis has also evolved from qualitatively determining the correlation between the results of the two histologies to calculating correlation coefficients using statistical analyses. Recently, Zhang et al. statistically analyzed the differential genes and metabolites obtained from the transcriptome and metabolome and calculated the Pearson correlation coefficients between these genes and metabolites [87]. Their study demonstrated that phenazine-1-carboxylic acid (PCA) primarily affects functions related to mitochondrial steroid, lipid, sterol, and oxidoreductase activity, as well as pathways involving cofactors, steroids, porphyrins, and cytochromes. In addition to zebrafish, combined transcriptomic and metabolomic analyses have been used in other aquatic biotoxicology studies [88,89,90,91,92,93,94,95,96,97,98,99,100]. For instance, when looking into the dangers of sewage, the use of native species to test exposure and multi-omics to determine the levels of risk and check for biomolecular markers provide new means to test the local environment. Guo et al. used the native species *Hemiculter leucisculus* to assess the hazards of local phenolic compound-containing effluents using omics analyses and screened key molecules as biomarkers [101]. Combined transcriptome and metabolome analyses have also been used in freshwater organisms such as shellfish, shrimp, algae, and trevally.

Combined transcriptomic and metabolomic analyses have been less frequently used in toxicological studies of marine organisms, mainly focusing on fish, shrimp, and shellfish. In 2019, a study utilizing this approach explored the effects of ammonia exposure on the shrimp *Litopenaeus vannamei*, including the mechanisms of ammonia response and tolerance [102]. This study provides new insights into the molecular mechanisms resolving shrimp ammonia adaptation strategies. After this, studies on the harmful effects of pollutants on different species, like *Mytilus galloprovincialis*, *Sparus aurata*, *Tachypleus tridentatus*, *Dunaliella salina*, and others, slowly emerged. These studies are very useful for marine ecotoxicology studies.

The core value of transcriptome–metabolome integration lies in establishing causal linkages from gene regulation to metabolic phenotypes [103,104,105,106,107,108,109,110,111,112,113,114,115,116]. Fish, particularly zebrafish, serve as the optimal platform for this approach. Embryonic or adult fish tissues provide sufficient biomass (>100 mg) for the concurrent extraction of both transcriptomic (RNAs) and metabolomic (small molecules) components. Crucially, the conserved detoxification and metabolic pathways in fish (e.g., CYP450, PPARγ) exhibit high homology to those in mammalian systems, enabling the direct mechanistic elucidation of pollutant impacts through omics data. Furthermore, well-annotated databases for model fish like zebrafish (e.g., ZFIN, KEGG) facilitate the rapid correlation of dysregulated genes with perturbed metabolites, thereby enabling cross-omics biomarker validation [117,118,119,120,121]. Overall, the transcriptome and metabolome can complement each other well. The transcriptome can give the metabolome sources of changed metabolites, and the metabolome can give molecular information that is more in line with the phenotype. The combination of the two can successfully direct enriched biological pathways to potentially undesirable outcomes, providing a more reliable basis for assessing the molecular toxic effects of pollutants and screening for specific molecular markers. See Table 2.

### 4.4. Combination of Transcriptomics and Proteomics Analysis

The transcriptome focuses on the mRNAs from which genes are transcribed, while the proteome focuses on the expression and function of proteins. The combination of the two can be used to more comprehensively analyze the processes of genes from transcription to translation and reveal the whole picture of gene expression regulation. Due to the mature development of transcriptomics, the method of joint transcriptome and proteome analysis has also become a hotspot in research in some fields. In the field of aquatic toxicology, there have been studies to analyze the mechanisms of toxicity by this method, with most of them focusing on the study of freshwater organisms.

Among the studies on freshwater organisms, those that reveal the molecular mechanisms of toxicity through the multi-omics analysis of fish are the most numerous. As early as 2008, Wim’s team used zebrafish to assess the liver toxicity of tetrabromobisphenol A (TBBPA) [123]. Differentially expressed genes and differentially expressed proteins were analyzed by microarray transcriptomics and differential in-gel electrophoresis (DiGE) proteomics. In 2010, Martyniuk’s group investigated the neurotoxicity of environmentally relevant concentrations of Dieldrin on largemouth bass [52]. Transcriptomic and proteomic measurements and analyses of samples demonstrated that Dieldrin was capable of altering normal life activities at both the structural gene and protein levels and had an impact on cellular integrity and survival. In 2023, Xu, Ying explored the mechanisms of neurodevelopmental toxicity of tri(1,3-dichloropropyl) phosphate (TDCPP) via the transcriptome and proteome [124]. A rationale for the mechanism of TDCPP-mediated non-genomic toxicity was provided by the similar pathways that were highly enriched in the two omics results. Although the transcriptome and proteome appear to be closely linked, the presence of post-transcriptional regulatory mechanisms may cause inconsistencies between the two types of omics data. In Singh’s research, he used multi-omics techniques to study chemically induced inflammation in zebrafish. An analysis of the data revealed that only 44% of the genes showed changes consistent with the proteome at the transcript level, 38% of the genes showed no change, and 18% of the genes showed changes opposite to the proteome [125]. Following this, another study conducted basic transcriptomic and proteomic studies on the fathead minnow (*Pimephales promelas*) and generated tissue-specific omics data to support future aquatic ecotoxicology genomics and endocrine-related studies [126]. In the study, they also found that not all genes showed a positive correlation between RNA-seq and proteomics, suggesting that post-transcriptional regulation is more common in eukaryotes and that transcriptomic results cannot be equated with proteomic results. On the other hand, analyzing the two omics together can better reveal the biological processes by which the key regulatory genes or proteins lead to phenotypic changes. How to integrate and analyze these two extremely relevant but different types of data to obtain a multi-level systematic analysis has been the focus of many studies. Qiao et al. utilized ingenuity pathway analysis (IPA) to combine transcriptomic and proteomic results with classical pathway analysis, as well as molecular and cellular functional assays, to provide evidence for the specific mechanism of action of cyanobacterial toxin toxicity towards other aquatic organisms at low concentrations in aquatic environments [25]. Alcaraz considered that the detection of proteins would be limited by instrumental detection limits and chromatographic identification. Therefore, transcripts were filtered using the IDs of the major proteins of the proteome, and the Pearson correlation between transcript expression and protein abundance was calculated to establish the relationship between the transcriptome and the proteome and predict the neurotoxicity of fluoxetine (FLX) [23]. Toxicological studies with combined transcriptome and proteome analyses are now appearing in other freshwater organisms (shrimp, algae, grebes, etc.) [127,128,129]. The most commonly chosen strategy is to analyze the DEGs together with DEPs by GO and KEGG enrichment to construct gene–protein network relationships.

Among the studies on marine organisms, fewer transcriptomic and proteomic studies have been carried out, and these studies have focused on shellfish. Only neonicotinoid pesticide mixtures and the metal element cadmium have been studied at multiple molecular levels [130,131,132]. This can be explained by the fact that the genomes of many marine organisms have not been fully sequenced, making it difficult to determine the entire protein expression profile of an organism.

Current studies utilizing integrated transcriptome–proteome approaches in aquatic ecotoxicology exhibit a pronounced bias towards freshwater fish species. This predominance arises from the ample tissue availability, evolutionarily conserved detoxification pathways, and robust data integration capacity in fish. Crucially, transcriptome–proteome integration outperforms single-omics methods by capturing post-transcriptional dynamics and providing the dual-layer validation of biomarkers. Thus, fish serve as the optimal platform in reconstructing “gene-to-protein” toxicity cascades, although the expansion of marine applications requires overcoming sample heterogeneity and genomic annotation gaps.

### 4.5. Combination of Transcriptomics, Proteomics, and Metabolomics Analysis

Currently, researchers are becoming progressively less satisfied with analyzing the mechanisms of pollutant toxicity at the partial molecular level. Integrated transcriptomics, proteomics, and metabolomics analyses enable the comprehensive reconstruction of pollutant-induced molecular perturbations within organisms. In 2021, Lee analyzed the toxic effects of perfluorooctane sulfonate (PFOS) on zebrafish through a combined transcriptomics, proteomics, and metabolomics analysis, identifying pathways of axonal deformation, neuroinflammatory stimulation, and dysregulation of calcium ion signaling [133]. In the same year, using a similar approach, they found that low concentrations of organochlorine pesticide mixtures were able to affect mitochondrial function, insulin signaling, and pathways related to energy homeostasis [134]. This approach broadens the molecular understanding of specific signaling pathways involved in the toxic effects of pollutants beyond that achieved with single-omics. Recently, Song’s team investigated the mechanisms of toxicity of ambient ultraviolet radiation (UVB) using three omics [135]. Notably, they used Crassostrea gigas, an organism that has not been used as a model, along with integrative network analysis (xMWAS) to connect the omics data obtained with biological functions or processes. Their study presents a new methodological approach to the integration and utilization of multi-omics data in non-model organisms. Currently, the most used integration methods for the joint analysis of the three omics are those proposed by Uppal in xMWAS v0.55, using sparse partial least squares (sPLS) regression for the integration and correlation analysis of the three types of omics data [136]. xMWAS performs network visualization, providing the topological distribution of nodes and multi-level community detection algorithms to identify highly connected nodes representing important molecules. Despite the many advantages of combined transcriptomics, proteomics, and metabolomics analyses, they are not currently used in a large number of studies due to the high costs and complexity associated with having three omics.

## 5. Conclusions and Future Prospects

Currently, different omics are more commonly used in various toxicological studies, with their high-throughput characteristics enabling the accurate and rapid localization of the molecular toxicological mechanisms of pollutants. The inherent complexity of cellular regulatory pathways, which involve diverse biomolecular entities and exhibit non-linear interdependencies, cannot be comprehensively captured through single-omics approaches. Such reductionist methodologies inevitably overlook substantial biological variations, whereas multi-omics integration enables the direct detection of the majority of pathway responses to chemical exposure. Recent years have witnessed growing interest in multi-omics applications within aquatic toxicology. The expanded implementation of these approaches is imperative to leverage the available genomic resources in aquatic organisms for high-throughput quantification, enhanced data reproducibility, and the discovery of critical biomarkers. A fundamental limitation in current aquatic studies lies in the insufficient characterization of conserved pathways and network interactions, which hampers the reconstruction of coherent molecular landscapes from multi-omics outputs. This deficiency often manifests as low concordance between findings derived from distinct omics analyses. Current mitigation strategies emphasize prioritizing consistency across omics measurements to align results with underlying biological realities, while acknowledging the absence of universally optimal analytical methodologies.

Microbiome alterations precede metabolomic changes, enabling the discovery of earlier and more sensitive biomarkers through multi-omics approaches in toxicological research. However, advancements in developing robust multi-biomarker panels through multi-omics platforms require deeper investigations into species-specific biological mechanisms and ecological relevance within aquatic systems. This requires establishing ecotoxicological sentinel model species beyond conventional laboratory organisms to better reflect environmental exposure scenarios.

In multi-omics experimental design, the strategic selection of omics layers must be guided by research objectives and model system characteristics. Investigators should employ prior chemical knowledge and pathway databases to identify critical biomolecules and signaling cascades, thereby determining appropriate omics technologies. The comprehensive analysis of AOPs inherently demands multi-tiered omics integration—for instance, metabolomics for hormonal perturbations, proteomics for enzymatic alterations, and transcriptomics for non-coding RNA or transcription factor dynamics. Methodological choices should therefore be hypothesis-driven to fully elucidate AOP components. Temporal dynamics and dose-specific responses warrant particular consideration, given that most published aquatic ecotoxicological multi-omics studies rely on limited exposure concentrations or duration endpoints.

Overall, multi-omics has brought innovative tools and new challenges to toxicology. On the one hand, it is necessary to integrate omics into traditional toxicology. The pathology and biochemical index experiments of traditional toxicology are corroborated with omics, so that the microscopic molecular toxicity mechanism can explain the macroscopic pathological features. On the other hand, it is necessary to improve the processing and mining of the large amounts of data acquired by high-throughput tools through machine learning and other methods.

## Figures and Tables

**Table 2:** Application of joint transcriptome and metabolome analysis for aquatic toxicology studies.

Number	Biological Group	Species	Contaminant	Reference
		Freshwater organisms		
1	Fish	*Danio rerio*	Di-(2-ethylhexyl) phthalate	[117]
2	Fish	*Danio rerio*	Geosmin	[118]
3	Fish	*Danio rerio*	Tebuconazole, difenoconazole	[119]
4	Fish	*Danio rerio*	Acetaminophen, diphenhydramine, carbamazepine, and fluoxetine	[120]
5	Fish	*Danio rerio*	Triclosan and its derivative, methyl-triclosan	[121]
6	Fish	*Danio rerio*	Bisphenol A	[80]
7	Fish	*Danio rerio*	Mefentrifluconazole	[84]
8	Fish	*Danio rerio*	Tire wear particles, road particles	[122]
9	Fish	*Danio rerio*	Manganese	[85]
10	Fish	*Danio rerio*	Prochloraz	[81]
11	Fish	*Danio rerio*	Difenoconazole Tebuconazole	[82]
12	Fish	*Danio rerio*	Difenoconazole	[83]
13	Fish	*Danio rerio*	Phenazine-1-carboxylic acid	[87]
14	Fish	*Danio rerio*	Bisphenol A Tetrabromobisphenol A	[86]
15	Fish	*Cyprinus carpio*	MS-222 and 2-PE	[88]
16	Fish	*Cyprinus carpio*	Silver nanoparticles	[89]
17	Fish	*Carassius auratus*	Di-(2-ethylhexyl) phthalate	[90]
18	Fish	*Hemiculter leucisculus*	Phenolic compounds	[101]
19	Fish	*Monopterus albus*	Copper	[91]
20	Fish	*Nile tilapia*	Microcystin-LR	[92]
21	Shellfish	*Pomacea canaliculata*	Arsenic	[93]
22	Crab	*Eriocheir sinensis*	Aflatoxin B1	[94]
23	Algae	*Chlamydomonas reinhardtii*	Cadmium	[95]
24	Algae	*Raphidocelis subcapitata*	Tylosin	[96]
25	Daphnia	*Calanus finmarchicus*	Alkanolamines	[97]
26	Daphnia	*Daphnia magna*	Pyrene, fluoranthene	[98]
27	Daphnia	*Daphnia pulex*	Fullerene crystals (nC(60))	[99]
28	Daphnia	*Daphnia magna*	Cadmium	[100]
		Marine organisms		
29	Fish	*Gasterosteus aculeatus*	1,2:5,6-Dibenzanthracene	[79]
30	Fish	*Gasterosteus aculeatus*	Ethinyl-estradiol	[103]
31	Fish	*Sparus aurata*	N,N-Diethyl-3-methyl benzoyl amide	[104]
32	Fish	*Sparus aurata*	Sulisobenzone	[105]
33	Fish	*Salmo salar*	Vitamin E, chlorpyrifos	[106]
34	Prawn	*Litopenaeus vannamei*	Ammonia	[102]
35	Prawn	*Litopenaeus vannamei*	Ammonia	[107]
36	Prawn	*Litopenaeus vannamei*	Microplastics, di-(2-ethylhexyl) phthalate	[108]
37	Shellfish	*Mytilus galloprovincialis*	Graphene nanomaterials, triphenyl phosphate	[109]
38	Shellfish	*Mytilus edulis*	Perfluorooctanoic acid	[110]
39	Shellfish	*Chlamys farreri*	Inorganic arsenic	[111]
40	Shellfish	*Mactra veneriformis*	Progestins	[112]
41	Shellfish	*Uditapes philippinarum*	Mercury, benzo(a)pyrene	[113]
42	Shellfish	*Strongylocentrotus purpuratus*	Polyvinyl chloride microplastics	[114]
43	Crab	*Tachypleus tridentatus*	Cadmium	[115]
44	Algae	*Dunaliella salina*	Cadmium	[116]

## Data Availability

Data availability is not applicable to this article as no new data were created or analyzed in this study.

## Appendix A

**Table A1:** Third-generation sequencing platforms.

Sequencing Platform	Reads	Accuracy
Single-Molecule Real-Time (SMRT)	10~25 kp	97%
Single-Molecule Nanopore	1000~4500 bp	87% (Read Mode), 99% (Accuracy Mode)
True Single Molecular Sequencing (tSMS)	100 kb	96%
Fluorescence Resonance Energy Transfer (FRET)	>1500 bp	>99%

**Table A2:** Comprehensive transcriptomic technologies in aquatic toxicology.

Technology	Principle	Aquatic Application Advantages
Bulk RNA-Seq		
Illumina Short-Read	NGS of fragmented RNA	Gold standard for DEG analysis High sensitivity for low-abundance transcripts in fish gills
PacBio Iso-Seq	Long-read full-length cDNA	Resolves complex splice variants in crustaceans No assembly needed
Oxford Nanopore	Direct RNA sequencing	Real-time mRNA surveillance Detects RNA modifications in live algae
Single-Cell		
10× Genomics	Barcoded droplet partitioning	Cell-type resolution in zebrafish embryos Immune cell profiling
Smart-seq2	Full-length scRNA-seq	High sensitivity for rare cell types
Spatial		
Visium (10×)	Spatial barcoding on slides	Maps pollutant gradients in fish liver lobules Correlates histopathology with gene expression
MERFISH	Multiplexed FISH imaging	Single-molecule resolution in biofilm communities Quantifies horizontal gene transfer

**Table A3:** Comprehensive proteomic technologies in aquatic toxicology.

Technology	Advantages	Disadvantages
Mass Spectrometry		
MALDI-TOF	Rapid analysis, tolerant to salt/washers	Limited resolution (<50,000 *m*/*z*), poor detection of low-abundance proteins
Orbitrap	Ultra-high resolution (>150,000 *m*/*z*), low detection limit	High instrument cost, strict desalination required for high-salt samples
Separation Techniques		
Gel-based (2D-PAGE)	Intuitive separation of post-translationally modified proteins	Low recovery for hydrophobic/membrane proteins (<30%), fails in high-salt conditions
Non-gel-based (LC-MS)	Enhanced peak capacity, improved detection of low-abundance proteins	Surface-active agents may interfere with chromatographic separation
Quantitative Methods		
Labeled (TMT)	16-channel simultaneous quantification, relative error <10%	High reagent cost (USD 500/sample), limited channel number
Label-free (LFQ)	Unlimited sample throughput, cost reduction (60%)	Poor reproducibility (CV > 20%), high missing value rate (>15%)
